# Comprehensive analysis of the survival outcomes and causes of death among patients diagnosed with myeloid sarcoma in the United States from 2000 to 2016: A retrospective SEER-based study

**DOI:** 10.1097/MD.0000000000041112

**Published:** 2025-01-03

**Authors:** Bahaa Mali, Ali Mali, Alaa Mali, Mohammad Hammad, Mohammed Abdulrazzak, Afnan W.M. Jobran

**Affiliations:** a Faculty of Medicine, Al Quds University, Jerusalem, Palestine; b Faculty of Medicine, University of Aleppo, Aleppo, Syria

**Keywords:** causes of death, myeloid sarcoma, relative survival, SEER

## Abstract

Myeloid sarcoma (MS) is a rare hematological malignancy characterized as an extramedullary tumor mass of neoplastic myeloid blasts that may involve various anatomical sites and affect their tissue structure. Given that MS is very rare, there is insufficient knowledge regarding its clinical features and no well-established therapeutic guidelines. We conducted a retrospective study of MS patients diagnosed between 2000 and 2016 using the Surveillance, Epidemiology, and End Results (SEER) database. We studied survival outcomes across different demographic and therapeutic subgroups. We also investigated the causes of death among our aimed cohort of patients. We found that between 2000 and 2016, 573 MS cases were reported in SEER 17 registries. Most patients were males (57.9%), and 55.1% were 60 or older. Most were non-Hispanic white (67.7%) and married (47.8%). Almost 61.4% were diagnosed with MS as their first primary tumor and 51.3% had only 1 tumor. In terms of treatment, 51.1% received chemotherapy, 26.2% underwent radiation therapy, and 13.6% had surgical management. The relative survival rate for MS patients in the United States is quite low, with a 3-year relative survival rate of 43.4%, declining to 39.0% at 5 years. Treatment with chemotherapy or surgical management has shown better survival outcomes. The primary cause of death is malignant diseases, particularly leukemias. Most deaths occur within the first year of diagnosis, with the risk gradually declining over time. MS is a rare malignant disease with a poor prognosis. Age and tumor location at diagnosis are important factors affecting survival. Chemotherapy is the most common treatment and has been found to improve survival. Most deaths in MS cases are due to malignant diseases, particularly leukemias. Future prospective studies are required to provide more significant outcomes and create targeted management regimens to enhance survival.

## 1. Introduction

Myeloid sarcoma (MS) is a rare hematological malignancy characterized as an extramedullary tumor mass of neoplastic myeloid blasts that may involve various anatomical sites and affect their tissue structure.^[1,2]^ According to a previous study based on Surveillance, Epidemiology, and End Results (SEER) data, the age-adjusted incidence rate of MS in the United States peaked at 0.077 per 100,000 persons in 2015, with male patients having a significantly higher incidence than females.^[3]^

Based on the World Health Organization’s (WHO) classification, MS is a rare subtype of acute myeloid leukemia (AML).^[4]^ This high-grade malignancy can occur simultaneously with, follow, or precede the diagnosis of intramedullary AML or other myeloid malignancies such as myelodysplastic syndrome, myeloproliferative neoplasm, or chronic myelogenous leukemia.^[5]^

Given that MS is very rare, there is insufficient knowledge regarding its clinical features and no clear therapeutic guidelines. This study aims to gain an in-depth understanding of the relative survival outcomes across different demographic and therapeutic subgroups. The study also seeks to investigate the causes of death among patients with MS to give future insights that might enhance the care and long-term follow-up strategies for this uncommon disease.

## 2. Methods

We identified patients diagnosed with MS in the United States between 2000 and 2016 using the most recent version of the SEER*Stat program^[6]^, 8.3.9.2. The MS histology code (code 9930/3) from the International Classification of Diseases for Oncology, third edition, was used to identify these cases using the SEER 17 registry database, which was released in April 2024 and based on the November 2023 submission.^[6]^

We extracted the patient’s data, including age, sex, race and ethnicity, marital status, year of diagnosis, total number of tumors per patient, first primary tumor indicator, primary location of involvement, and therapy received. The primary site of involvement for MS patients was divided into 11 groups: bone, breast, connective and soft tissues, chest and abdomen, digestive system, head and neck, hematopoietic and reticuloendothelial systems, nervous system, pelvis and genitourinary organs, skin, and unknown. After analyzing the data with the Jamovi program, we have presented descriptive statistics to reveal the epidemiological and clinical characteristics of our cohort.

We utilized the SEER program’s survival session to determine the 3-year and 5-year relative survival rates for patients diagnosed with MS in the United States between 2000 and 2016. The relative survival rate, as defined by the SEER, is a measure of net survival that represents cancer survival in the absence of other causes of death. We calculated the ratio of observed to expected survival while adjusting for the general survival rate of the US population based on race, sex, age, and the date when the age was coded.

To investigate the causes of death, we utilized the SEER Cause of Death Recode, which was established based on the WHO International Classification of Diseases 10th Revision (ICD-10).^[7]^ We assessed each cause of death using the standardized mortality ratio (SMR) and their 95% confidence intervals (CIs) at different survival intervals. These intervals were categorized as less than 1 year, between 1 and 5 years, between 5 and 10 years, and more than 10 years. To calculate SMR, SEER compared the actual number of deaths among MS patients to the number of deaths that would be expected in patients of similar age and demographics during the same time frame. A statistically significant *P* value was considered to be less than 0.05.

We adhered to the “Strengthening the Reporting of Observational Studies in Epidemiology” guidelines while presenting the findings of this research. We did not require approval from the institutional review board because we obtained our research cohort data from the SEER registry, which is publicly accessible and anonymized.

## 3. Results

Between 2000 and 2016, a total of 573 cases of MS were identified in SEER 17 registries in the USA. We found that 58.1% of these cases were diagnosed in and after 2009, while 41.9% were diagnosed before. The cohort showed a male predominance, with 57.9% being male and 55.1% being 60 years old or older. Most of these cases were non-Hispanic white, accounting for 67.7% of the cases, followed by Hispanic at 14.5%. In terms of marital status, 47.8% of patients were married, 26.2% were single, 8.4% were widowed, and 8.0% were divorced or separated (Table 1).

**Table 1:** Baseline characteristics of patients diagnosed with myeloid sarcoma from 2000 to 2016, based on the SEER database

Variable	Count	%
Total	573	100
Age group (years)		
<40	133	23.2
40–59	124	21.6
≥60	316	55.1
Sex		
Male	332	57.9
Female	241	42.1
Marital status		
Single	150	26.2
Married	274	47.8
Widowed	48	8.4
Divorced/separated	46	8.0
Unknown	55	9.6
Year of diagnosis		
2000–2008	240	41.9
2009–2016	333	58.1
Race		
NHW	388	67.7
NHB	51	8.9
NHAPI	43	7.5
Hispanic	83	14.5
NHAIAN	3	0.5
NHUR	5	0.9
First primary tumor		
Yes	352	61.4
No	221	38.6
Number of tumors		
One	294	51.3
Two or more	279	48.7
Surgery		
Yes	78	13.6
No/unknown	495	86.4
Chemotherapy		
Yes	293	51.1
No/unknown	280	48.9
Radiation		
Yes	150	26.2
No/unknown	423	73.8

NHAIAN **=** Non-Hispanic American Indian/Alaska Native, NHAPI **=** Non-Hispanic Asian or Pacific Islander, NHB **=** Non-Hispanic Black, NHUR **=** Non-Hispanic Unknown Race, NHW **=** Non-Hispanic White.

In our patient cohort, 352 individuals (61.4%) were diagnosed with MS as their first primary tumor, while 221 patients (38.6%) had a previous primary tumor. Among our patients, 51.3% (294 individuals) had only 1 tumor, while 48.7% (279 individuals) had 2 or more tumors per patient. Regarding treatment, 51.1% received chemotherapy, 26.2% underwent radiation therapy, and 13.6% received surgical management (Table 1). We observed that the most frequent primary sites at the time of presentation were connective and soft tissues (31.6%), hematopoietic and reticuloendothelial systems (17.8%), and skin (11.0%) (Table 2).

**Table 2:** Counts and percentages of patients diagnosed with myeloid sarcoma from 2000 to 2016 based on the primary site of presentation in the SEER database.

Primary Site	Counts	(n%)
Bone	30	5.2
Breast	10	1.7
Chest and abdomen	34	5.9
Connective and soft tissues	181	31.6
Digestive system	52	9.1
Head and neck	39	6.8
Hematopoietic and reticuloendothelial systems	102	17.8
Nervous system	19	3.3
Pelvis and genitourinary organs	33	5.8
Skin	63	11.0
Unknown	10	1.7

Our analysis indicates that individuals in the United States with MS have a relatively low relative survival rate. The rate is 43.4% after 3 years and declines to 39.0% after 5. For male patients, the 3-year relative survival rate is 45.4%, whereas for female patients, it is 40.8%. Patients under the age of 40 have a 3-year relative survival rate of 63.8%, patients between 40 and 59 have a rate of 50.5%, and patients over 60 have a rate of 24.7%. Comparing the 3-year relative survival rates by race, non-Hispanic Whites had 38.3%, non-Hispanic Blacks 41.2%, and non-Hispanic Asians or Pacific Islanders 52.3%. The 3-year relative survival rates for Hispanics were higher at 60.2%. Three-year survival for non-Hispanic American Indian/Alaska Native patients with MS was not achieved, nevertheless, by any of the 3 individuals included. Regarding marital status, widowed patients had a reduced 3-year relative survival rate of 12.6%. Single patients had a rate of 53.3%, married patients had a rate of 40.6%, divorced or separated patients had a rate of 47.9%, and patients with unknown marital status had a rate of 49.7% (Table 3).

**Table 3:** Three-year and 5-year relative survival rates among patients diagnosed with myeloid sarcoma from 2000 to 2016, based on the SEER database.

Variable	Relative survival % at 3 years	Relative survival % at 5 years
Total	43.4 (37.9–48.8)	39.0 (33.4–44.4)
Age group (years)		
<40	63.8 (52.6–73.0)	58.0 (46.7–67.7)
40–59	50.5 (39.0–60.9)	45.9 (34.6–56.5)
≥60	24.7 (17.9–32.1)	21.2 (14.5–28.8)
Sex		
Male	45.4 (38.0–52.5)	40.9 (33.4–48.3)
Female	40.8 (32.6–48.8)	36.4 (28.4–44.4)
Race		
NHW	38.3 (31.8–44.8)	32.9 (26.4–39.5)
NHB	41.2 (21.8–59.7)	37.4 (18.8–56.1)
NHAIAN	0.0%	0.0%
NHAPI	52.3 (32.1–69.1)	52.3 (32.1–69.1)
Hispanic	60.2 (45.5–72.1)	56.4 (41.7–68.8)
NHUR	75.1 (12.7–96.1)	75.1 (12.7–96.1)
Marital status		
Single	53.3 (42.8–62.7)	49.0 (38.6–58.7)
Married	40.6 (32.8–48.3)	37.5 (29.6–45.3)
Widowed	12.6 (3.1–28.9)	0.0
Divorced/separated	47.9 (28.1–65.3)	41.1 (21.9–59.3)
Unknown	49.7 (28.2–68.0)	42.6 (22.1–61.7)
Surgery		
Yes	54.9 (40.5–67.2)	47.5 (33.2–60.6)
No/unknown	41.1 (35.1–46.9)	37.0 (31.1–42.9)
Chemotherapy		
Yes	51.1 (43.0–56.8)	43.8 (36.8–50.6)
No/unknown	32.6 (24.3–41.2)	31.5 (22.8–40.6)
Radiation		
Yes	41.8 (31.5–51.8)	34.0 (24.2–44.0)
No/unknown	44.0 (37.5–50.4)	40.7 (34.1–47.1)

CI **=** confidence interval, NHAIAN **=** Non-Hispanic American Indian/Alaska Native, NHAPI **=** Non-Hispanic Asian or Pacific Islander, NHB **=** Non-Hispanic Black, NHUR **=** Non-Hispanic Unknown Race, NHW **=** Non-Hispanic White.

Our results showed that patients who underwent surgery had a higher 3-year and 5-year relative survival rate (54.9% and 47.5%, respectively) compared to those who did not undergo surgery or whose status was unknown (41.1% and 37.0%, respectively). Furthermore, patients who received chemotherapy also exhibited higher 3-year and 5-year relative survival rates (51.1% and 43.8%, respectively) compared to those who did not receive chemotherapy or whose status was unknown (32.6% and 31.5%, respectively). However, we did not observe a similar improvement in relative survival among patients who received radiotherapy (Table 3).

During the follow-up period, 240 patients died after being diagnosed with MS. Out of these, 187 (77.92%) died because of cancer diseases, and 53 (22.08%) died of noncancer diseases. The risk of mortality among our cohort was higher than that of the general USA population (SMR, 11.80; 95% CI, 10.36–13.39). The majority of deaths (155; 64.58%) occurred within the first year following MS diagnosis. Additionally, 60 (25%) deaths occurred within 1 to 5 years following MS diagnosis, and 25 (10.42%) deaths occurred after more than 5 years (Table 4).

**Table 4:** Causes of death among patients diagnosed with Myeloid sarcoma from 2000 to 2016, based on the SEER database.

	<1 year		1–5 years		5–10 years		>10 years		Total	
	Obs.	SMR (95% CI)	Obs.	SMR (95% CI)	Obs.	SMR (95% CI)	Obs.	SMR (95% CI)	Obs.	SMR (95% CI)
All Causes of Death	155	41.29* (35.05– 48.33)	60	7.60* (5.8– 9.78)	16	3.05* (1.74– 4.95)	9	2.62* (1.2– 4.97)	240	11.80* (10.36–13.39)
All Malignant Cancers	131	146.29* (122.32–173.6)	44	25.91* (18.83–34.79)	8	7.23* (3.12– 14.25)	4	5.45* (1.48– 13.95)	187	42.18* (36.35–48.67)
Non-Hodgkin Lymphoma	7	207.74* (83.52–428.02)	1	15.79 (0.4–87.96)	0	0 (0– 93.28)	0	0 (0– 141.21)	8	49.17* (21.23–96.88)
Leukemias	109	2987.29* (2452.88– 3603.56)	42	593.59* (427.81– 802.36)	7	157.20* (63.2–323.88)	3	98.62* (20.34– 288.22)	161	883.68* (752.45– 1031.21)
Myeloid and Monocytic Leukemia	93	5224.51* (4216.85– 6400.38)	38	1106.24* (782.84– 1518.40)	6	262.40* (96.29– 571.13)	3	184.95* (38.14– 540.49)	140	1534.44* (1290.80– 1810.70)
Acute Myeloid Leukemia	49	3393.02* (2510.17– 4485.75)	31	1111.30* (755.08– 1577.40)	4	212.58* (57.92– 544.3)	3	224.69* (46.34– 656.65)	87	1167.72* (935.29– 1440.38)
Acute Monocytic Leukemia	2	11,133.48* (1348.32– 40,217.95)	0	0 (0–11,609.19	0	0 (0–20,534.87)	0	0 (0 ––31,676.99	2	2520.52* (305.25– 9104.99)
Chronic Myeloid Leukemia	2	1103.36* (133.62– 3985.73)	0	0 (0– 1085.27)	2	958.04* (116.02– 3460.76)	0	0 (0– 2583.19)	4	458.33* (124.88– 1173.51)
Other Myeloid/Monocytic Leukemia	40	29,261.03* (20,904.51– 39,845.22)	7	2556.14* (1027.70– 5266.62)	0	0 (0–2069.05)	0	0 (0–2784.33)	47	6515.77* (4787.54 – 8664.59)
Other Leukemia	16	1872.49* (1070.29– 3040.81)	4	256.15* (69.79– 655.84)	1	109.35* (2.77– 609.29)	0	0 (0 – 639.8)	21	537.49* (332.71– 821.6)
Non–Hematologic Malignancy	15	18.62* (10.42–30.72)	1	0.66 (0.02– 3.65)	1	1 (0.03–5.58)	1	1.51 (0.04– 8.42)	18	4.51* (2.67–7.13)
Non-cancer causes	24	8.40* (5.38–12.49)	16	2.58* (1.48–4.19)	8	1.93 (0.83–3.80)	5	1.85 (0.60– 4.32)	53	3.33* (2.50–4.36)
In situ, benign or unknown behavior neoplasm	5	206.54* (67.06–482.00)	4	80.80* (22.01–206.87)	0	0 (0–120.86)	0	0 (0–178.01)	9	72.02* (32.93–136.72)
Infectious causes	1	6.78 (0.17–37.79)	1	3.32 (0.08–18.5)	0	0 (0–20.03)	1	8.72 (0.22–*48.59)	3	4.01 (0.83–11.73)
Cardiovascular diseases	7	6.38* (2.57– 13.15)	3	1.32 (0.27– 3.86)	4	2.81 (0.77–7.19)	0	0 (0–4.13)	14	2.46* (1.35–4.13)
Cerebrovascular Diseases	2	9.35* (1.13–33.79)	0	0 (0–8.6)	1	3.64 (0.09–20.29)	1	6.53 (0.17–36.36)	4	3.74* (1.02–9.57)
Accidents and Adverse Effects	1	7.54 (0.19– 42.03)	3	9.29* (1.92–27.16)	0	0 (0–14.81)	1	5.49 (0.14–30.59)	5	5.64* (1.83–13.16)
Other non–cancer causes	8	6.64* (2.86–13.08)	5	1.83 (0.59–4.26)	3	1.57 (0.32–4.6)	2	1.56 (0.19–5.64)	18	2.52* (1.5–3.99)

Obs = observed, SMR **=** standardized mortality ratio.

* Indicates *P* value less than 05.454 y.

Leukemia is the leading cause of cancer-related deaths. A total of 161 patients died due to leukemia, resulting in an SMR of 883.68 (95% CI, 752.45–1031.21). The majority of deaths due to leukemia occurred within the first year after an MS diagnosis (109 patients, 67.70%), followed by 1 to 5 years after the diagnosis (42 patients, 26%). The SMR for leukemia-related deaths decreased gradually over time. Most deaths were due to myeloid and monocytic leukemia (140 patients), with AML being the most common subtype (87 patients) (Table 4).

The risk of dying from nonhematologic cancer is over 4 times higher in the MS population compared to the general USA population (SMR, 4.51; 95% CI, 2.67–7.13), and most deaths occur within the first year following MS diagnosis. The highest causes of noncancer-related deaths are cardiovascular diseases (14 patients, SMR, 2.46; 95% CI, 1.35–4.13), followed by in situ, benign, or unknown behavior neoplasms (9 patients, SMR, 72.02; 95% CI, 32.93–136.72), accidents and adverse effects (5 patients, SMR, 5.64; 95% CI, 1.83–13.16), and cerebrovascular diseases (4 patients, SMR, 3.74; 95% CI, 1.02–9.57) (Table 4).

## 4. Discussion

Our study revealed that MS is a rare hematological malignancy. We identified only 573 patients in the SEER 17 registries in the USA between 2000 and 2016. Among these cases, 58.1% were diagnosed in and after 2009, while 41.9% were diagnosed before. The study found that 57.9% of the patients were male, with 55.1% of them being 60 years old or older. Additionally, 67.7% were of non-Hispanic white race, and 47.8% were married. These findings align with a previous SEER registry-based study, which showed that the rate of MS in the United States peaked at 0.077 per 100,000 persons in 2015, with males having a significantly higher incidence than females.^[3]^ Consistent with the demographics of our study, prior registry-based studies reported that the median age at MS diagnosis was 59 years, and white race predominance.^[8,9]^

MS is a rare hematological malignancy that can occur alongside, follow, or precede the diagnosis of intramedullary AML or other myeloid malignancies.^[5,10,11]^ In our study, we observed that patients with MS as the initial and sole tumor were more common, accounting for nearly half of the patient population. Given the rarity of the disease, there are no established guidelines for managing MS. However, it is typically treated with AML chemotherapy regimens, and surgery and radiotherapy may be used as adjuvant therapy.^[12–15]^ In our study, 51.1% of patients received chemotherapy, 26.2% underwent radiation therapy, and 13.6% underwent surgical treatment.

According to our analysis, individuals in the United States with MS have a relatively low relative survival rate. The rate is 43.4% after 3 years and declines to 39.0% after 5 years. The relative survival rates were predominantly lower among patients older than 60 years, females, non-Hispanic Whites, and patients with widowed marital status. Other authors report that the age and primary site of involvement are independent prognostic factors. Patients with disease presenting in the nervous system, lymph nodes, or hematopoietic tissue had the lowest survival rates, while gender or race didn’t affect overall survival.^[3,9,15]^

In terms of treatment, patients who underwent chemotherapy or surgical management showed better survival outcomes than those who did not. However, this improvement in survival was not observed among patients who received radiotherapy. However, due to the small number of patients who underwent surgery and radiotherapy, we should be cautious in concluding how treatment affects prognosis. Chemotherapy has long been recognized as an independent prognostic factor, and patients who received chemotherapy had a significantly higher survival rate than those who did not.^[16,17]^ Furthermore, other studies have indicated positive survival outcomes when chemotherapy is combined with radiotherapy or surgery.^[15,18]^ Goyal et al^[8]^ reported that early chemotherapy had no effect on overall survival in younger patients but was associated with higher mortality in older patients.

Our analysis of the causes of death among MS patients revealed that the majority of deaths were due to malignant diseases, particularly leukemias. Most deaths were attributed to myeloid and monocytic leukemia, with AML being the most common subtype. The majority of deaths occurred within the first year of diagnosis, followed by the period between the first year and fifth year after diagnosis, with a gradual decline in the risk over time. These findings align with previous observations indicating that MS may co-occur with, follow, or precede the diagnosis of intramedullary AML or other myeloid malignancies, highlighting the importance of early chemotherapy.^[5,16,17]^

Death due to noncancer causes is also higher among MS patients. The leading noncancer cause of death is cardiovascular disease, followed by in situ, benign, or unknown behavior neoplasms, accidents and adverse effects, and cerebrovascular diseases. These findings emphasize the importance of modifying risk factors, early detection, and proper management of these conditions to further enhance survival.

While our cohort data was obtained from the SEER database, a high-quality registry with a rigorous quality assurance program that is particularly useful for studying the epidemiological and clinical features of rare malignancies, we need to acknowledge certain limitations. First, the retrospective cohort design of our study may increase the risk of bias in the study results. Second, the SEER database only provides information on early treatment and lacks information related to consolidation and maintenance therapy. Additionally, we did not have access to complete patient data, such as comorbidities and socioeconomic status, which could impact the survival outcomes.

## 5. Conclusion

MS is a rare malignant disease with a poor prognosis. Age and the location of the tumor at diagnosis are important factors that affect survival rates, with older patients over 60 and certain tumor-presenting sites such as the nervous system, lymph nodes, or hematopoietic tissue associated with lower survival outcomes. Chemotherapy is the most commonly used treatment and has been found to improve survival. The majority of deaths in MS cases are due to malignant diseases, particularly leukemias. Most deaths are attributed to myeloid and monocytic leukemia, with AML being the most common subtype, highlighting the importance of early chemotherapy. Prospective research in the future is required to provide more significant outcomes. To create targeted management regimens that may enhance survival, it is imperative to research the molecular, genetic, and pathophysiological basis of these cancers.

## Author contributions

**Conceptualization:** Bahaa Mali, Ali Mali, Alaa Mali, Afnan W.M. Jobran.

**Data curation:** Bahaa Mali, Ali Mali, Afnan W.M. Jobran.

**Formal analysis:** Bahaa Mali, Ali Mali, Afnan W.M. Jobran.

**Investigation:** Bahaa Mali, Ali Mali, Mohammad Hammad.

**Project administration:** Bahaa Mali, Afnan W.M. Jobran.

**Resources:** Bahaa Mali, Ali Mali, Alaa Mali, Mohammad Hammad, Afnan W.M. Jobran.

**Software:** Bahaa Mali, Ali Mali, Alaa Mali.

**Validation:** Bahaa Mali, Ali Mali, Alaa Mali, Mohammed Abdulrazzak.

**Writing—original draft:** Bahaa Mali, Ali Mali, Alaa Mali, Mohammad Hammad, Mohammed Abdulrazzak, Afnan W.M. Jobran.

**Writing—review & editing:** Bahaa Mali, Ali Mali, Alaa Mali, Mohammad Hammad, Mohammed Abdulrazzak, Afnan W.M. Jobran.

**Supervision:** Mohammed Abdulrazzak, Afnan W.M. Jobran.
